# Prevalence of Pulmonary Hypertension in Individuals With Heart Failure: A Systematic Review and Meta‐Analysis

**DOI:** 10.1002/clc.70197

**Published:** 2025-08-29

**Authors:** Maaedah Khan, Rhea Suribhatla, Jak Spencer, Nadia Daniel, Alex Pitcher, Christiana Kartsonaki

**Affiliations:** ^1^ Medical Sciences Division University of Oxford Oxford UK; ^2^ The Heart Centre John Radcliffe Hospital, Oxford University Hospitals NHS Foundation Trust Oxford UK; ^3^ Radcliffe Department of Medicine University of Oxford Oxford UK; ^4^ Clinical Trials Service Unit and Epidemiological Studies Unit (CTSU), Nuffield Department of Population Health University of Oxford Oxford UK

**Keywords:** heart failure, hypertension, meta‐analysis, prevalence, pulmonary, systematic review

## Abstract

**Aims:**

Heart failure (HF) is a leading cause of hospitalizations worldwide. HF can lead to pulmonary hypertension (PH) and co‐occurrence of HF and PH is associated with a poor prognosis. This systematic review and meta‐analysis aim to estimate the prevalence of PH in patients with HF.

**Methods:**

We searched MEDLINE and EMBASE for studies reporting the prevalence of PH amongst HF patients. A meta‐analysis of PH prevalence, including subgroup analyses, was conducted using a random‐effects model. Subgroup analyses and meta‐regressions by comorbidities and patient characteristics were done. Study quality was assessed using the Joanna Briggs Institute Critical Appraisal Tool.

**Results:**

Fifty‐four papers with 259 665 HF patients were included, of which 46 004 also had PH. The overall PH prevalence estimate in individuals with HF is 46.6% (95% CI: 39.6%–53.7%). Prevalence varied by diagnostic method, with studies using right heart catheterization reporting the highest estimates (62.5%; 52.0%–72.0%), hospital recorded data the lowest (18.4%; 14.4%–23.3%), and echocardiography 45.7% (37.1%–54.6%). Prevalence was higher in HF with preserved (47.2%; 34.8%–60.0%) than reduced ejection fraction (35.7%; 22.6%–51.3%). Prospective studies show higher estimates (60.1%; 50.7%–68.8%) than retrospective studies (37.3%; 29.5%–45.9%).

**Conclusions:**

This is the first systematic review and meta‐analysis investigating the prevalence of PH in HF patients and shows that the prevalence of PH in this patient population is strikingly high. There is notable variability in estimates reported by different studies, largely attributed to differences in the diagnostic method of PH. Future studies with robust, standardized methodologies are needed to estimate prevalence more accurately.

## Graphical abstract

This systematic review has shown that the prevalence of pulmonary hypertension among individuals with heart failure is high, and varies by several population characteristics, potentially reflecting patient selection, disease severity, and differences in diagnosis and treatment.

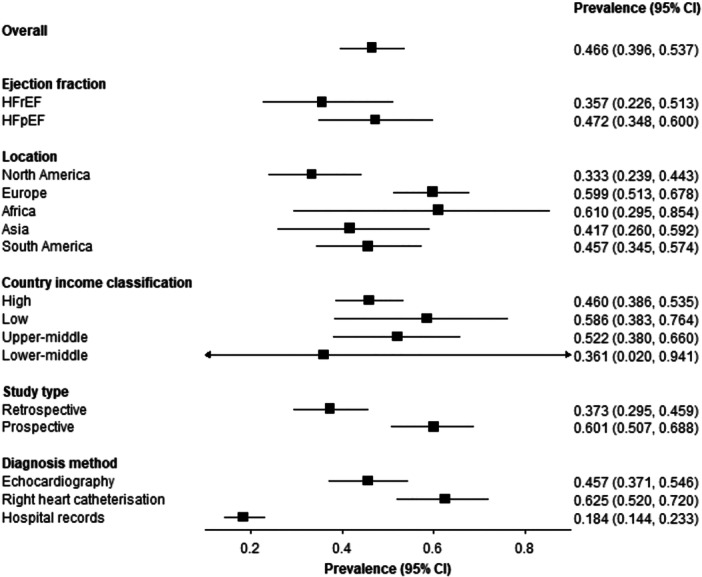

## 1 Introduction

The prevalence of pulmonary hypertension (PH) is currently reported as 12–53 cases per million people [1]. PH rates are increasing, particularly in resource‐limited areas, and two epidemics have been described in the last 60 years [2]. Thus, the disease is becoming an increasingly serious global health burden. The prognostic outcomes of PH patients are poor, with strikingly high mortality rates; the 1‐year mortality rate in the United States was estimated to be 8%, and 38% in high‐risk patients [3]. Early disease identification and intervention are key in reducing the significant morbidity and mortality associated with PH. This, coupled with new findings from large patient cohorts, has recently led to changes in the definition of PH, with the diagnostic threshold being lowered to a mean pulmonary artery pressure of 20 mmHg, instead of 25 mmHg [3, 4].

Transthoracic echocardiography is an important noninvasive, relatively low‐cost investigation to estimate PH probability. A higher peak tricuspid regurgitant velocity increases the probability of PH, for example, a velocity of > 3.4 m/s confers a high probability of PH in the absence of other features of PH on echocardiography [3]. However, right heart catheterization (RHC), which is a more invasive investigation, is the gold standard diagnostic method for PH. During RHC, pulmonary vascular resistance (PVR) and pulmonary artery wedge pressure (PAWP) can be measured. These measurements are important to differentiate between precapillary PH (PAWP ≤ 15 mmHg and PVR > 2 Wood units), postcapillary PH (PAWP > 15 mmHg and PVR > 2 Wood units), and combined precapillary and postcapillary PH (PAWP > 15 mmHg and PVR ≤ 2 Wood units) [3]. Precapillary PH results from remodeling of the pulmonary vasculature, whilst postcapillary PH results from increased pressure in the pulmonary vasculature due to cardiac disease [3].

PH has many causes which can be broadly split into five categories: idiopathic (Group I), left cardiac disease (Group II), lung disease (Group III), pulmonary artery obstruction (Group IV), and hematological/systemic disorders (Group V). Of these, heart failure (HF) is the most common cause of PH [5]. HF is the inability of the heart to adequately pump blood and meet the metabolic demands of the body. It is classified into three distinct groups, each with differing levels of ejection fraction (preserved, mid‐range, and reduced) [6]. The immense prevalence, mortality, and public health burden has even been described as a “global pandemic,” with reported prevalence rates ranging between 3.9 and 16 per 1000 people [1, 7, 8].

PH is a poor prognostic indicator in HF and is associated with an increased 5‐year mortality rate, independent of HF severity and other comorbidities [9]. These poor outcomes can be largely attributed to the lack of specific treatments available for Group II PH, in comparison to other PH subtypes.

Although the disease mechanisms linking HF and PH are well documented in the literature, little work has been done to establish the epidemiological relationship between the two pathologies in patients. This systematic review and meta‐analysis summarizes the literature investigating the co‐existence of these two pathologies, with a focus on the prevalence of PH in individuals with HF. The aims of this review are to investigate the overall prevalence of PH amongst individuals with HF, and to estimate how this varies across different populations and study characteristics, including the subtype of HF, presence of other comorbidities, and study methodology.

## 2 Methods

In this systematic review and meta‐analysis, MEDLINE and EMBASE were searched electronically on October 7, 2022, to find any papers related to PH and HF published since inception of the databases (1946). Key terms such as “heart failure”, “pulmonary hypertension”, and “prevalence” were included. The full search strategy of Medline and Embase is included in Supporting Information S1: Table 1. We included prospective, cross‐sectional, or retrospective studies on humans, in which individuals with HF were included and information on PH was mentioned. Studies exclusively on children (< 18 years of age), studies in a language other than English, or review articles were excluded.

Search results were uploaded to Covidence, and duplicates were removed. Two independent reviewers completed title/abstract (J.S., N.D.) and then full‐text screening (J.S., M.K./R.S.), according to the prespecified Prospero protocol CRD42023335272. Conflicts were resolved after discussion with another reviewer (C.K.).

Data extraction was completed in Covidence, using a template predesigned according to the Prospero protocol, by two independent reviewers (J.S., M.K./R.S.). Extracted data were consolidated with any discrepancies resolved by discussion. We extracted data on study characteristics (title, author, year of publication, location, special population, study design [retrospective/prospective], total number of participants, inclusion and exclusion criteria), study population (mean age, proportion female, ethnicity, mean BMI, proportion with pulmonary disease, obstructive sleep apnea, diabetes, anemia, kidney disease, smoking, atrial fibrillation, and ischemic heart disease), HF (number of participants with HF, diagnostic method, definition, subtype of HF [reduced/preserved ejection fraction]), and PH (number of participants with PH, number of participants with HF who also had PH, diagnostic method, definition). Overall and subgroup‐specific prevalence estimates were collected from each paper, if available. Countries were grouped based on their income using the World Bank Income classification.

A meta‐analysis of PH prevalence was done using a logit transformation and a random effects model, and as a sensitivity analysis using an inverse‐variance weighted average. If an overall and subgroup‐specific prevalence was extracted from a study, data from the latter was excluded in the overall analysis and only included in the relevant subgroup analysis. Prevalence estimates and 95% confidence intervals (CI), overall and in subgroups, based on a random effects model, are reported unless stated otherwise. Heterogeneity was assessed using *I* ^2^ and Cochran's *Q* test. Correlations between prevalence of PH and population characteristics (such as demographics and comorbidities, HF features, or etiology) were assessed graphically and using meta‐regression.

The papers included were critically appraised by two independent reviewers (M.K., R.S.) using the Johanna Briggs Institute Critical Appraisal Tool for systematic reviews (Supporting Information S1: Table 2) [10]. Data analysis was done using R version 4.2.2 and package “meta”.

## 3 Results

### 3.1 Study Selection

**Figure 1:**
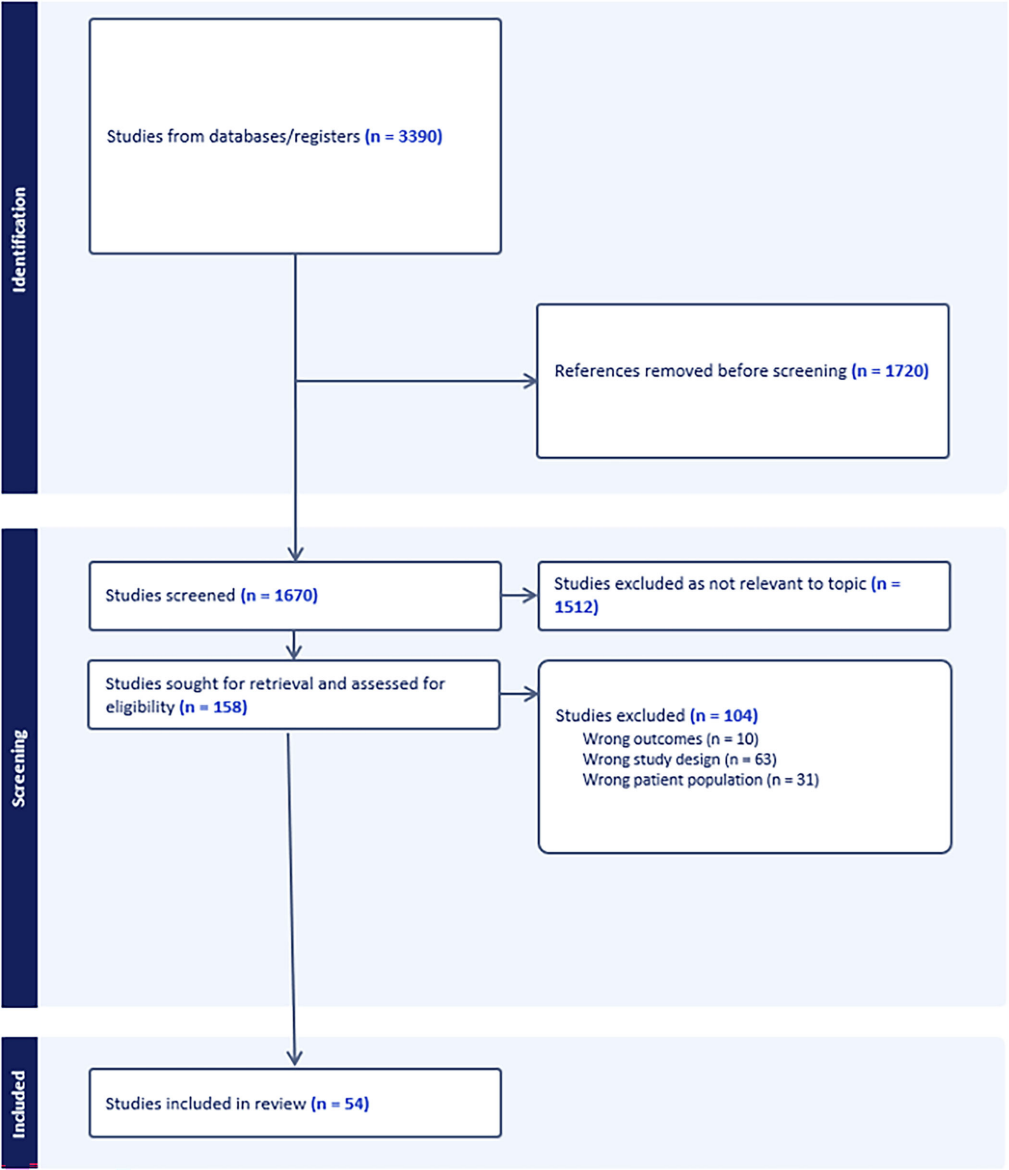
A summary of the study selection process.

Of the 3390 studies identified by the search strategy, following the removal of duplicates, 1670 full papers were screened. Overall, 54 papers were identified for inclusion in this review (Figure 1).

### 3.2 Characteristics of the Included Studies

Table 1 shows the characteristics of the studies included. The complete data set extracted from the studies is shown in Supporting Information S1: Table 3. Overall, 259 665 participants with HF were included, of which 46 004 also had a diagnosis of PH. The total number of participants within each study ranges from 10 to 188 991 [11, 12]. Overall, studies from 25 different countries were included, although most studies were conducted in the United States [12, 13, 14, 15, 16, 17, 18, 19, 20, 21, 22, 23, 24, 25, 26, 27, 28]. The methodology and patient demographics of the studies vary greatly, and patient data were collected from a variety of sources, including registries [12, 17, 29, 30, 31, 32, 33]. The reported mean age and BMI from the included studies range from 49.5 to 92.33 years and 22.7 to 32.6, respectively [27, 34, 35, 36].

**Table 1:** Main characteristics of included studies.

Paper	Location	Location per income	Study type	Group	HFrEF versus HFpEF	*N*	PH prevalence	PH diagnosis
Ahmadi 2021	North America (Canada)	High	Retrospective	Right ventricular dysfunction		10	60.00%	Echo
Ahmadi 2021	North America (Canada)	High	Retrospective	Normal right ventricular function		23	13.00%	Echo
Ahmed 2020	Europe (Sweden)	High	Prospective	Pre‐heart transplant		26	73.10%	RHC
Allison 2016	Europe (England)	High	Retrospective	Nonagenarians (overall)		144	47.00%	Echo
Allison 2016	Europe (England)	High	Retrospective	Nonagenarians (HFrEF)	HFrEF	63	50.79%	Echo
Allison 2016	Europe (England)	High	Retrospective	Nonagenarians (HFpEF)	HFpEF	55	32.73%	Echo
Alqahtani 2019	North America (USA)	High	Retrospective	Not for palliative care		188 991	14.90%	Hospital recorded data
Alqahtani 2019	North America (USA)	High	Retrospective	For palliative care		2338	28.30%	Hospital recorded data
Amadi 2016	Africa (Nigeria)	Low	Prospective	Overall		125	70.40%	Echo
Arnaert 2021	Europe (Belgium)	High	Retrospective	Overall		248	41.10%	Echo
Auffret 2020	Europe (France)	High	Retrospective	Post‐TAVR		102	77.20%	Echo
Benito‐Gonzalez 2020	Europe	High	Retrospective	HFrEF with fMR	HFrEF	93	14.00%	Echo
Bosch 2017	Asia (Singapore)	High	Prospective	HFrEF	HFrEF	219	52.00%	Echo
Bosch 2017	Asia (Singapore)	High	Prospective	HFpEF	HFpEF	219	39.00%	Echo
Butler 1999	North America (USA)	High	Retrospective	Overall	HFrEF	320	19.00%	RHC
Chakraborty 2022	North America (USA)	High	Retrospective	Hypertrophic cardiomyopathy		6040	30.40%	Hospital recorded data
Choudhary 2014	North America (USA)	High	Retrospective	Overall		107	74.80%	Echo
Covella 2017	North America (USA)	High	Retrospective	Hypertrophic obstructive cardiomyopathy		172	50.60%	RHC
Diaconu 2015	Europe (Romania)	High	Retrospective	Diabetes		67	19.40%	Hospital recorded data
Faggiano 2000	Europe (Italy)	High	Prospective	Aortic stenosis		216	69.90%	RHC
Gerges 2015	Europe (Austria)	High	Retrospective	Retrospective: Systolic HF		664	68.80%	RHC
Gerges 2015	Europe (Austria)	High	Retrospective	Retrospective: Diastolic HF		399	59.40%	RHC
Gerges 2015	Europe (Austria)	High	Prospective	Prospective: Systolic HF		172	80.80%	RHC
Gerges 2015	Europe (Austria)	High	Prospective	Prospective: Diastolic HF		219	76.70%	RHC
Guazzi 2017	Europe (Italy)	High	Prospective	HFpEF TAPSE/PASP ratio < 0.35	HFpEF	129	91.50%	Echo
Guazzi 2017	Europe (Italy)	High	Prospective	HFpEFTAPSE/PASP ratio 0.35–0.57	HFpEF	129	85.71%	Echo
Guazzi 2017	Europe (Italy)	High	Prospective	HFpEF TAPSE/PASP ratio > 0.57	HFpEF	129	81.81%	Echo
Haddad 2022	North America (USA)	High	Retrospective	HFpEF	HFpEF	96	55.00%	Echo
Hsieh 2016	Asia (Taiwan)	High	Retrospective	CKD requiring hemodialysis		160	31.90%	Echo
Huang 2022	Asia (China)	Upper‐middle	Prospective	HF and coronary artery disease		182	77.50%	RHC
Huang 2022	Asia (China)	Upper‐middle	Prospective	HFpEF and coronary artery disease	HFpEF	142	82.50%	RHC
Huang 2022	Asia (China)	Upper‐middle	Prospective	HFrEF and coronary artery disease	HFrEF	40	76.10%	RHC
Ibe 2016	Asia (Japan)	High	Retrospective	Overall		164	40.00%	RHC
Jentzer 2022	North America (USA)	High	Retrospective	Coronary care unit patients		2651	79.10%	Echo
Kanumuri 2019	Asia (India)	Lower‐middle	Retrospective	Hospitalized patients		130	4.60%	Echo
Karaye 2013	Africa (Nigeria)	Low	Prospective	Overall		80	66.25%	Echo
Khush 2009	North America (USA)	High	Retrospective	Overall	HFrEF	171	47.00%	RHC
Kushimo 2019	Africa (Nigeria)	Low	Prospective	Overall		219	38.80%	Echo
Lam 2009	North America (USA)	High	Prospective	HFpEF	HFpEF	203	83.00%	Echo
Lee 2022	Asia (South Korea)	High	Retrospective	Overall		1729	21.60%	Echo
Leung 2010	North America (USA)	High	Retrospective	HFpEF	HFpEF	455	68.80%	RHC
Lima 2019	North America (USA)	High	Retrospective	Overall		5595	3.04%	Hospital recorded data
Lin 2022	Asia (China)	Upper‐middle	Retrospective	Overall		480	44.80%	RHC
Liu 2020	Asia (China)	Upper‐middle	Prospective	HFpEF	HFpEF	149	39.60%	Echo
Lutsey 2022	North America (USA)	High	Retrospective	Venous thromboembolism patients		6189	22.40%	Hospital recorded data
Mogollon 2008	Europe (Spain)	High	Retrospective	Pre‐heart transplant		39	48.70%	RHC
Mutlak 2018	Asia (Israel)	High	Prospective	fTR		709	72.00%	Echo
Nakagawa 2020	South America (Brazil)	Upper‐middle	Prospective	HFrEF	HFrEF	70	46.00%	Echo
Nakamura 2019	Asia (Japan)	High	Prospective	HFpEF	HFpEF	198	46.50%	RHC
Nkoke 2022	Africa (Cameroon)	Lower‐middle	Prospective	Overall		66	93.90%	Echo
Pandey 2020	North America (USA)	High	Retrospective	Hospitalized with HFrEF in 2005–2009	HFrEF	5440	12.20%	Hospital recorded data
Pandey 2020	North America (USA)	High	Retrospective	Hospitalized with HFrEF in 2010–2014	HFrEF	7883	12.10%	Hospital recorded data
Pandey 2020	North America (USA)	High	Retrospective	Hospitalized with HFpEF in 2005–2009	HFpEF	4278	13.20%	Hospital recorded data
Pandey 2020	North America (USA)	High	Retrospective	Hospitalized with HFpEF in 2010–2014	HFpEF	7335	20.70%	Hospital recorded data
Pintalhao 2017	Europe (Portugal)	High	Retrospective	Overall		117	59.80%	Echo
Raina 2015	North America (USA)	High	Retrospective	Overall		537	59.60%	RHC
Raina 2015	North America (USA)	High	Prospective	Octogenarians with aortic stenosis		36	80.60%	RHC
Rifaie 2010	Africa (Egypt)	Lower‐middle	Prospective	Overall	HFpEF	100	20.00%	Echo
Santas 2019	Europe (Spain)	High	Prospective	Overall	HFpEF	760	77.80%	Echo
Selvaraj 2017	North America (USA)	High	Prospective	CKD		35	22.90%	Echo
Shah 2014	North America (USA)	High	Retrospective	HFpEF	HFpEF	935	36.00%	Echo
Sobieszczanska‐Malek 2014	Europe (Poland)	High	Retrospective	Overall		559	66.70%	RHC
Stein 2012	Asia (Israel)	High	Retrospective	Overall		9335	29.50%	Echo
Straburzynska‐Migaj 2007	Europe (Poland)	High	Prospective	Overall	HFrEF	56	41.00%	Echo
Torres‐Macho 2012	Europe (Spain)	High	Retrospective	Overall		419	62.30%	RHC
Vanhercke 2015	Europe (Belgium)	High	Prospective	Overall		401	69.80%	Echo
Vanhercke 2015	Europe (Belgium)	High	Prospective	HFrEF	HFrEF		67.00%	Echo
Vanhercke 2015	Europe (Belgium)	High	Prospective	HFpEF	HFpEF		73.00%	Echo
VanWezenbeek 2022	Europe (Netherlands)	High	Retrospective	HFpEF	HFpEF	46	71.70%	RHC
Wang 2014	Asia (China)	Upper‐middle	Retrospective	HFrEF requiring CRT	HFrEF	183	50.30%	Echo
Zotter‐Tufaro 2015	Europe (Austria)	High	Prospective	HFpEF	HFpEF	174	85.10%	RHC

Abbreviations: CKD = chronic kidney disease; CRT = cardiac resynchronisation therapy; fMR = functional mitral regurgitation; fTR = functional tricuspid regurgitation; PASP = pulmonary artery systolic pressure; TAPSE = tricuspid annular plane systolic excursion; TAVR = transcatheter aortic valve replacement.

The definitions and diagnostic methods for PH were highly heterogeneous. Thirty‐nine papers formally reported their definition for diagnosing PH [13, 16, 17, 18, 19, 20, 21, 25, 27, 28, 29, 30, 31, 32, 33, 35, 36, 37, 38, 39, 40, 41, 42, 43, 44, 45, 46, 47, 48, 49, 50, 51, 52, 53, 54, 55, 56, 57, 58]. The majority of papers used pulmonary arterial pressure with cut‐offs ranging from > 25 to > 45 mmHg [16, 19, 20, 21, 25, 28, 29, 30, 31, 33, 35, 36, 37, 38, 40, 41, 42, 43, 44, 45, 46, 48, 49, 50, 51, 52, 53, 54, 55, 56, 57, 58]. Echocardiography [11, 15, 17, 18, 20, 26, 27, 29, 31, 32, 34, 35, 37, 39, 42, 45, 47, 48, 50, 51, 52, 53, 54, 56, 57, 59, 60, 61, 62, 63] RHC [13, 16, 19, 21, 25, 28, 30, 33, 36, 38, 40, 41, 43, 44, 46, 49, 55, 58] were the most commonly used methods for diagnosing PH in the included studies.

Generally, HF was more thoroughly assessed and defined, with the New York Heart Association classification or the Framingham criteria being the most used assessment tools. Seventeen papers focused exclusively on patients requiring hospitalization for HF [12, 14, 15, 19, 24, 25, 29, 32, 38, 39, 42, 48, 51, 54, 56, 59, 64]. Some studies calculated prevalence estimates partially and/or exclusively on special HF patient cohorts such as those with valvular disease [28, 49, 54, 59, 60], chronic kidney disease [26, 37], or hypertrophic cardiomyopathy [14, 16].

One study by Lima et al. focused exclusively on post‐partum women; although we did not specify pregnancy/post‐partum as an exclusion criterion in our protocol, we felt that this cohort did not represent the general HF population given its distinct pathophysiology, and thus excluded this study from the main analysis [22].

### 3.3 Primary Outcome

**Figure 2:**
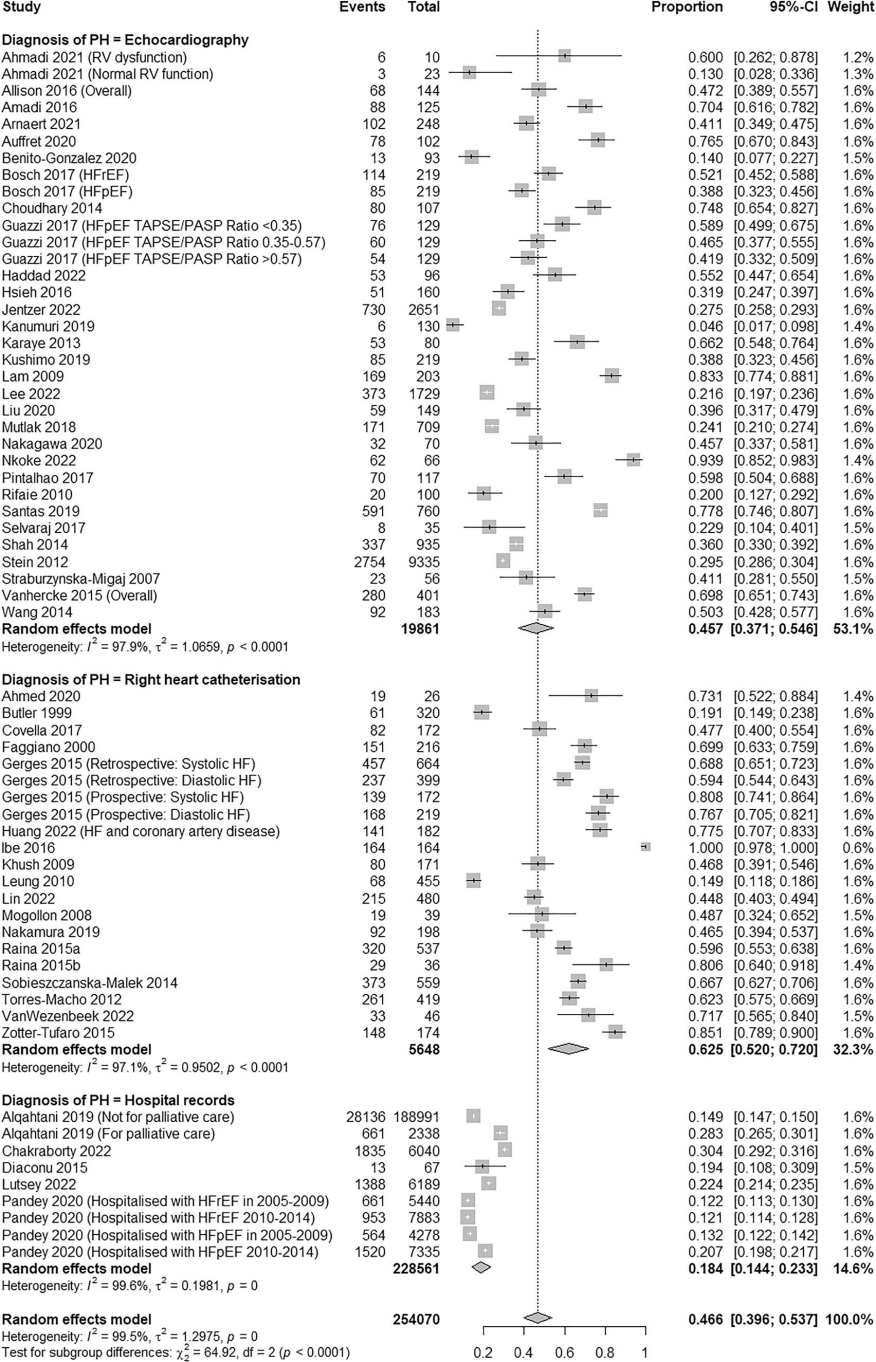
A forest plot showing the overall prevalence of pulmonary hypertension amongst those in heart failure and the prevalence as per diagnostic method (echocardiography, right heart catheterization, and hospital‐recorded data).

The overall PH prevalence amongst individuals with HF was 46.6% (95% CI: 39.6%–53.7%) based on the random effects model (Figure 2). The reported prevalence in the included studies ranges from 4.6% [61] to 93.9% [56] (Table 1, Figure 2). The former estimate arises from a retrospective analysis carried out in 130 hospitalized patients in India, whilst the latter is derived from a prospective study in 66 patients hospitalized with acute HF in Cameroon. An overall pooled estimate including Lima et al. is included in Supporting Information S1: Figure 1.

### 3.4 Secondary Outcomes

There was significant variability in the prevalence ranges which is expected due to the different inclusion criteria and settings of the included studies. We found that the main driver of this observed heterogeneity was attributed to the diagnostic method used. Studies were grouped based on their diagnostic method, and a sensitivity analysis was performed using the common effects model (Figure 2, Supporting Information S1: Figure 2). Thirty studies used echo to report PH prevalence estimates (*n* = 19 861) [12, 16, 18, 19, 21, 27, 28, 30, 31, 32, 33, 35, 36, 38, 39, 41, 43, 46, 48, 49, 52, 53, 54, 55, 57, 59, 60, 62, 63, 64], 18 used right heart RHC (*n* = 5648) [14, 17, 20, 22, 26, 29, 34, 40, 42, 44, 45, 47, 50, 51, 56, 58, 61, 65], and 5 used hospital recorded data (*n* = 228 561) [11, 12, 13, 14, 15, 16] (Table 1, Figure 2). Studies using RHC reported the highest PH prevalence estimates (62.5%; 52.0%–72.0%) amongst those with HF and those using hospital‐recorded data reported the lowest prevalence estimates (18.4%; 14.4%–23.3%). Studies using echo reported prevalence estimates of 45.7% (37.1%–54.6%). Although there was a small overlap between CI for the estimates reported using echo and RHC, the difference observed between these estimates is statistically significant (*p* = 0.0169).

**Figure 3:**
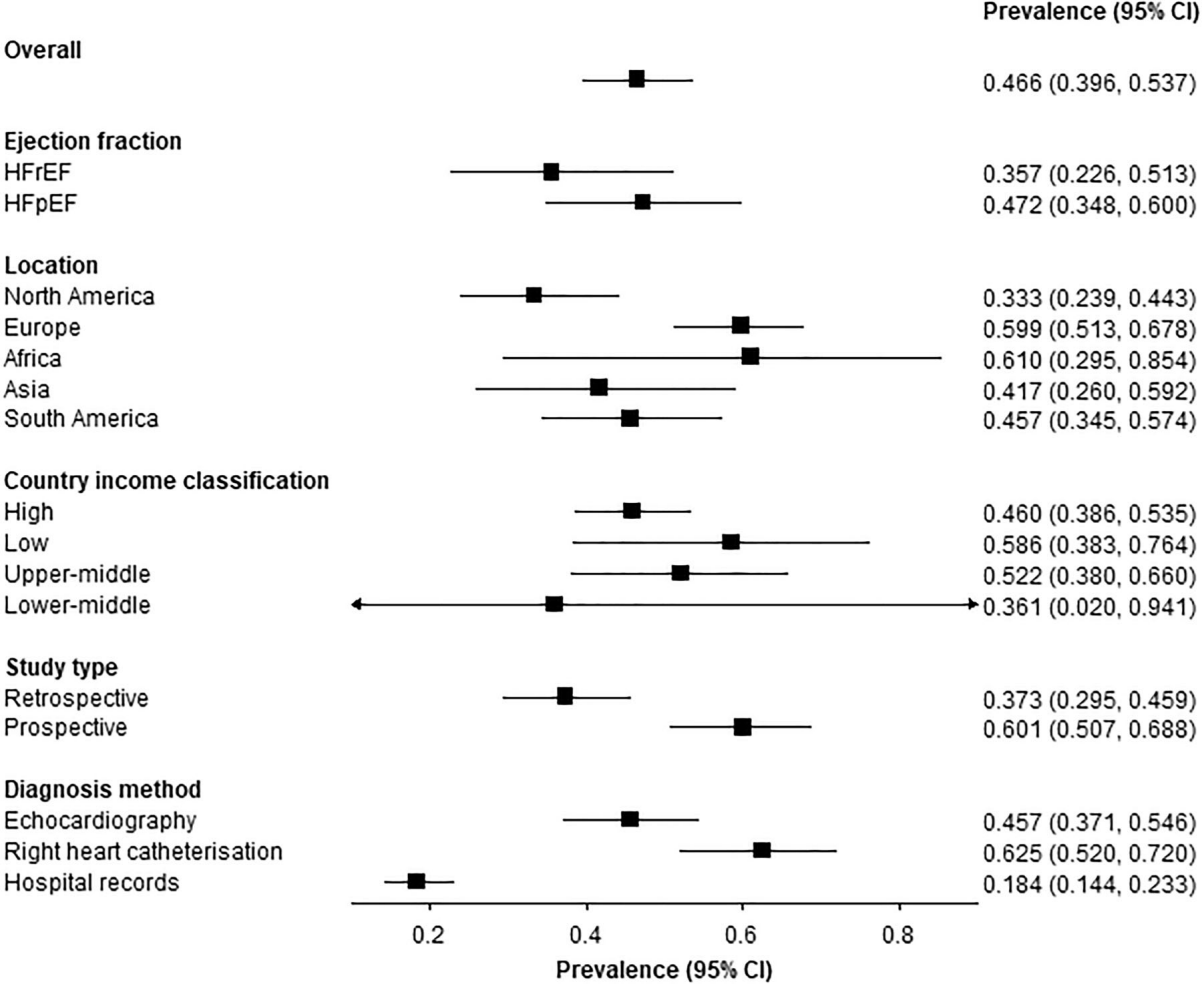
A forest plot showing how the prevalence of pulmonary hypertension amongst those with heart failure varies with ejection fraction, location, country income classification, study type, and diagnostic method.

Subgroup analysis of PH prevalence amongst adults with HF by ejection fraction, continental region, country income classification, and study methodology was performed (Figure 3). Of the investigated factors, only study design (retrospective vs. prospective) showed a significant difference in the reported prevalence estimates. Prospective studies have higher overall PH prevalences (60.1%; 50.7%–68.8%) in comparison to retrospective studies (37.3%; 29.5%–45.9%) (Figure 3, Supporting Information S1: Figure 3). Of the papers included in this review, 30 are retrospective [11, 12, 13, 14, 15, 16, 17, 18, 19, 21, 23, 24, 25, 27, 29, 34, 35, 36, 37, 38, 39, 40, 41, 42, 43, 44, 45, 59, 60, 61, 64], 21 are prospective [20, 25, 26, 30, 31, 32, 33, 46, 47, 48, 49, 50, 51, 52, 53, 54, 55, 56, 57, 62, 63], and 1 study had retrospective and prospective subgroups [58] (Table 1, Supporting Information S1: Figure 3).

**Figure 4:**
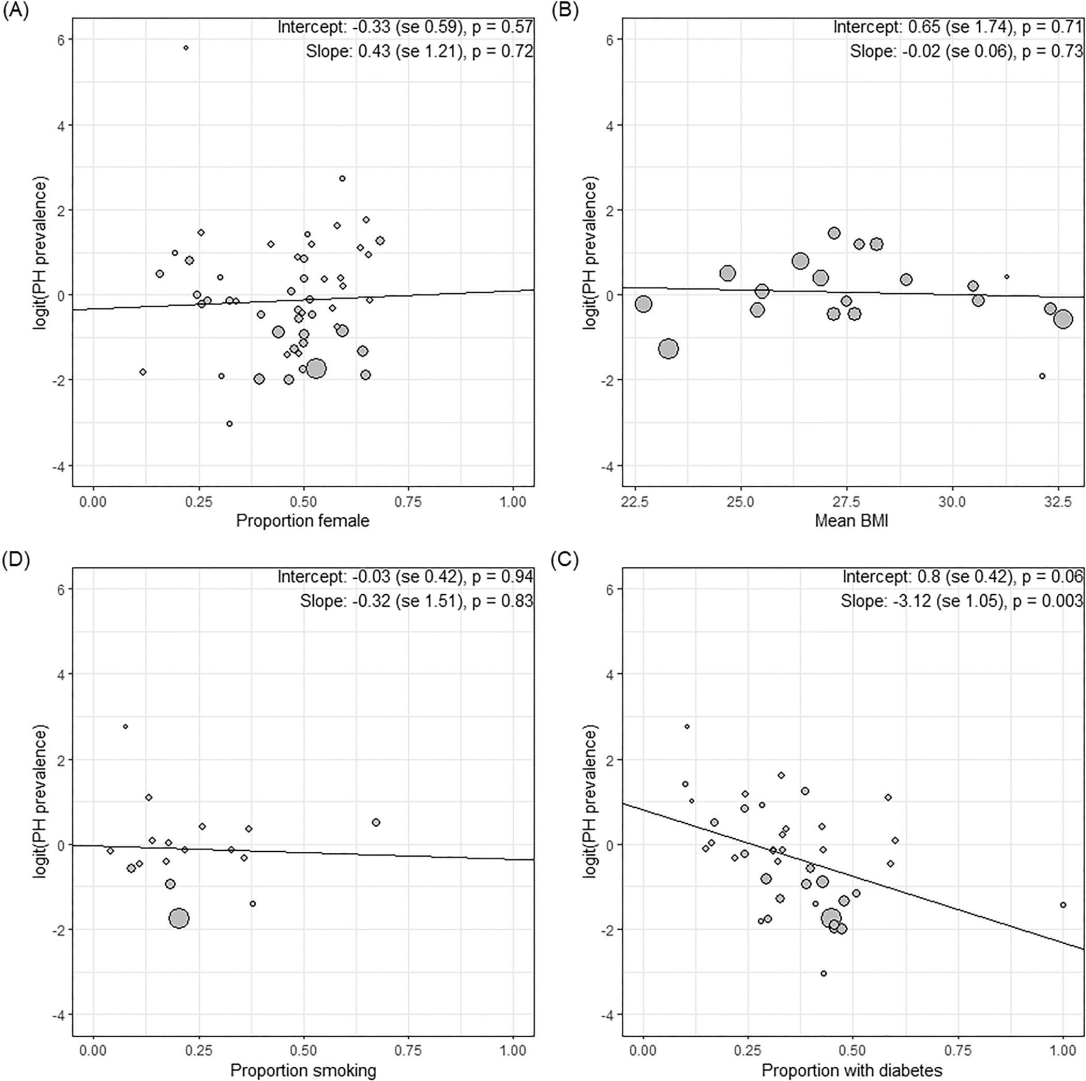
Meta‐regression analysis showing how the prevalence of pulmonary hypertension amongst those with heart failure varies with (A) proportion of female participants, (B) mean BMI, (D) proportion of participants who smoke, and (C) the proportion of participants who have diabetes.

Analysis based on study design and diagnostic method is detailed in Supporting Information S1: Figure 4. Studies using echo and RHC to diagnose PH used a mix of retrospective and prospective methodologies, whilst studies using hospital recorded data were exclusively retrospective in nature (Table 1, Supporting Information S1: Figure 4). Prevalence estimates reported by prospective studies remained higher than estimates reported by retrospective studies, even when split by diagnostic method.

Ten and 15 papers include PH prevalence data on patients with HF with reduced ejection fraction (HFrEF) [13, 19, 24, 30, 34, 45, 48, 60, 62, 63] and/or HF with preserved ejection fraction (HFpEF) [17, 20, 21, 24, 27, 30, 31, 33, 34, 44, 48, 50, 53, 55, 57], respectively (Table 1, Supporting Information S1: Figure 5). PH prevalence was lower in patients with HFrEF (35.7%; 22.6%–51.3%) than HFpEF (47.2%; 34.8%–60.0%) (Figure 3, Supporting Information S1: Figure 5). Analysis based on subtype of HF and diagnostic methods are shown in Supporting Information S1: Figure 6.

When grouping the studies into continental regions, North America [11, 12, 13, 14, 15, 16, 17, 18, 19, 20, 21, 23, 24, 25, 26, 27] and Europe [29, 31, 32, 33, 34, 35, 40, 41, 43, 44, 46, 49, 50, 58, 59, 60, 63, 64] account for most of the papers (17), followed by Asia (12) [30, 36, 37, 38, 39, 42, 45, 48, 53, 54, 55, 61], Africa (5) [47, 51, 52, 56, 57], and South America (1) [62] (Table 1, Supporting Information S1: Figure 7). Studies from North America had the lowest calculated prevalence of PH in HF (33.3%; 23.9%–44.3%) and studies from Africa had the highest (61.0%; 29.5%–85.4%) (Figure 3, Supporting Information S1: Figure 7).

Fourty‐two studies were conducted in high income countries [11, 12, 13, 14, 15, 16, 17, 18, 19, 20, 21, 23, 24, 25, 26, 27, 28, 29, 31, 32, 33, 34, 35, 37, 38, 39, 40, 41, 42, 43, 44, 46, 48, 49, 50, 54, 55, 58, 59, 60, 63, 64], 5 in upper‐middle [30, 45, 53, 62, 65], 3 in lower‐middle [56, 57, 61], and 3 in low income countries [47, 51, 52] (Table 1, Supporting Information S1: Figure 8). The highest estimate of PH amongst those with HF came from low‐income countries (58.6%; 38.3%–76.4%) and the lowest estimates came from lower‐middle income countries (36.1%; 2.0%–94.1%) (Figure 3, Supporting Information S1: Figure 8).

### 3.5 Other Outcomes

Meta‐regression showed that diabetes is the only investigated factor which shows a significant association with PH prevalence (slope estimate: −3.12; *p* < 0.003) (Figure 4D). The remaining factors, including smoking and BMI, were not associated with PH prevalence in this meta‐regression analysis (Figure 4, Supporting Information S1: Figure 9).

## 4 Discussion

PH remains a serious complication of HF and is associated with a significant increase in morbidity and mortality. To the best of our knowledge, this is the first systematic review and meta‐analysis investigating the prevalence of PH in individuals with HF. We included 54 papers with 259 665 participants to calculate the global estimate of PH prevalence amongst those with HF. Overall, we show that the prevalence of PH amongst those with HF is strikingly high; approximately of individuals with HF are at risk of developing this complication. Compared to the general population, individuals with HF are nearly 20 times more likely to develop PH [66]. Given that PH can significantly worsen the prognosis in HF, it is of utmost importance that HF patients are carefully monitored to detect early signs of PH to enable prompt interventions.

### 4.1 Heterogeneity

The included studies were highly heterogeneous in their methodology and patient characteristics. Our subgroup analysis shows that studies which use RHC, are prospective in nature, or have a higher proportion of patients with HFpEF report higher PH prevalence estimates. We show that the PH diagnostic method (RHC, echo, and hospital‐recorded data) is the main contributor to heterogeneity, and this finding is corroborated by our sensitivity analysis performed when studies were split by diagnostic method. Studies using RHC report the highest prevalence estimates, followed by echo and then hospital recorded data, which reports the lowest estimates. In this review, echo was the most commonly used method of diagnosing PH. It should be noted that patients diagnosed by RHC have more severe HF than those diagnosed by echo, presenting a source of inherent bias reflected by the higher PH estimates calculated using RHC.

In this review, six papers used hospital‐recorded data and the majority of these studies relied on ICD‐10 codes to confirm the PH diagnosis. Although hospital‐recorded data tends to be less reliable, these studies used large patient cohorts and may be more representative of a general HF cohort.

Another notable factor affecting prevalence is study methodology. In general, prospective studies are likely to be more reliable as investigators have more control over data collection and standardization of disease diagnosis. By following participants over time, prospective studies can also provide more comprehensive and accurate data of PH prevalence in HF. However, perhaps due to their large time‐ and resource‐intensity, the number of individuals with the outcome of interest in prospective studies is often small.

In this meta‐analysis, we show that the prevalence estimate reported by prospective studies is significantly higher than those reported by retrospective studies. This may be partly due to differences in diagnostic methods used in retrospective and prospective studies (e.g. hospital‐recorded data are exclusively used in retrospective studies). However, this does not appear to fully explain the difference, as prospective studies report higher prevalence estimates even when grouped by diagnostic method. This finding is mirrored by Greges et al., the only paper included in this review which uses retrospective and prospective patient groups, albeit to a lesser extent [58]. Here, the authors used RHC to measure the prevalence of PH in HF and reported that the prevalence was lower in retrospective cohorts (65.3% compared to 78.5%).

### 4.2 Geographical Location

We show that the continent with the lowest reported PH prevalence rate is North America, which is also the continent contributing to the highest number of papers in the review. Africa shows the highest prevalence, although it has very few data points. These findings may reflect global differences in PH diagnostic practices and HF treatments. Interestingly, studies using RHC to diagnose PH were exclusively carried out in high and/or upper‐middle‐income countries. RHC remains the gold standard for diagnosing PH but may be inaccessible in certain locations due to its invasive nature and cost. Further studies investigating PH prevalence using the gold‐standard diagnostic methods are needed, particularly in low‐income countries.

### 4.3 Subtype of HF

Our results show that HFpEF is associated with higher PH prevalence compared to HFrEF. This fits in with the underlying pathophysiology of PH, as HFpEF may lead to increased left atrium pressure and subsequent pulmonary venous congestion. Furthermore, there are very limited treatment options for HFpEF in comparison to what is available for HFrEF [66]. This, coupled with the fact that individuals with HFpEF are reported to have higher survival rates, means that such patients may be more likely to develop the long‐term sequelae of HF, which include PH.

### 4.4 Diabetes

The results of our meta‐regression analysis contradict other studies, which report higher rates of PH in diabetic patients [67, 68, 69]. Our result that diabetes prevalence is negatively associated with PH prevalence is counterintuitive and can likely be explained by the heterogeneity of studies included in our analysis and the ecological biases of study‐level associations. It could also represent a lack of screening for PH in this population.

### 4.5 Strengths and Limitations

This review has numerous strengths. First, it systematically and comprehensively synthesizes the global prevalence of PH in individuals with HF. Second, our thorough subgroup analysis highlights how different factors contribute to the observed heterogeneity between study prevalence estimates. Our use of the random effects model appropriately accounts for study heterogeneity in our meta‐analysis and enables us to provide an accurate overall estimate of PH prevalence. Third, we have included the common effects as a sensitivity analysis in the Supporting Information S1: Figures.

There are several limitations to this review. The included papers use a variety of mean pulmonary artery pressure thresholds to define PH; 35 mmHg was the most common, but definitions ranged between 25 and 45 mmHg. Also, some participants who may have only had a transient increase in pulmonary pressures due to congestion may have been included in the studies, rather than those with true PH. Similarly, multiple criteria were used to report HF disease diagnosis and severity, including the Framingham criteria and European Society of Cardiology Guidelines [5, 70]. Furthermore, our meta‐regression is limited by study‐level associations and further work is needed to investigate risk factors associated with developing PH in those with HF. Our search yielded one randomized controlled trial which was TOPCAT (Treatment of Preserved Cardiac Function Heart Failure with an Aldosterone Antagonist Trial). Although this could represent a limitation in our search strategy, it more likely reflects the lack of PH assessment in HF trials. Future HF trials should investigate PH development as an outcome, given the increased mortality associated with developing this complication.

## 5 Conclusion

Overall, the prevalence of PH in individuals with HF reported in the 54 included papers is strikingly high, with an estimate of 45.4% (95% CI: 38.0%–52.9%). There was marked heterogeneity between studies included in this meta‐analysis, and this was largely driven by differences in PH diagnostic methods. Studies using RHC, which is the gold‐standard diagnostic method, report higher PH prevalence estimates (62.5%; 52.0%–72.0%). Studies using hospital‐recorded data report the lowest PH prevalence (15.7%; 10.4%–22.9%) and those using echo report estimates of 45.7% (37.1%–54.6%). Moving forward, future studies should focus on harmonization and standardization of the methodology, diagnostic techniques, and definitions used to improve the accuracy of PH estimates in individuals with HF. In addition to this, further work is needed to investigate the risk factors associated with developing PH amongst those with HF to identify patient subgroups who may benefit from early intervention.

## Author Contributions

C.K. conceived and designed the study. J.S. carried out searches. J.S., N.D., M.K., R.S., and C.K. screened search results. J.S., M.K., and R.S. extracted data. C.K. and J.S. analyzed the data. M.K., R.S., J.S., A.P., and C.K. drafted the manuscript. All authors reviewed and edited the final draft. All authors had full access to all the data in the study and the final responsibility for the decision to submit for publication.

## Ethics Statement

The authors have nothing to report.

## Consent

The authors have nothing to report.

## Conflicts of Interest

The authors declare no conflicts of interest.

## Supporting information

Supplementary information.

Supplementary Table 3. Complete data extracted from the included studies.

## Data Availability

All data are available as supplementary information.
