# Supplementary material for: Prevalence of Pulmonary Hypertension in Individuals With Heart Failure: A Systematic Review and Meta‐Analysis
Source: Clin Cardiol. 2025 Aug 29;48(9):e70197. doi: 10.1002/clc.70197 (PMC12395587; doi:10.1002/clc.70197)

**Supplementary material**

**Prevalence of pulmonary hypertension in individuals with heart failure: a systematic review and meta-analysis**

Maaedah Khan^1*^, Rhea Suribhatla^1*^, Jak Spencer^1^, Nadia Daniel^1^, Alex Pitcher^2^, Christiana Kartsonaki^3^

^1^ Medical Sciences Division, University of Oxford

^2^The Heart Centre, John Radcliffe Hospital, Oxford University Hospitals NHS Foundation Trust, Oxford, UK; Radcliffe Department of Medicine, University of Oxford

^3^Clinical Trials Service Unit and Epidemiological Studies Unit (CTSU), Nuffield Department of Population Health, University of Oxford

^*^ Joint first authors

Address for correspondence:

Christiana Kartsonaki

CTSU

NDPH, Big Data Institute Building

University of Oxford

Old Road Campus

Oxford, OX3 7LF, UK

Tel: 44-1865-743626

Fax: 44-1865-743985

**Supplementary table 1. Search strategy**

Embase


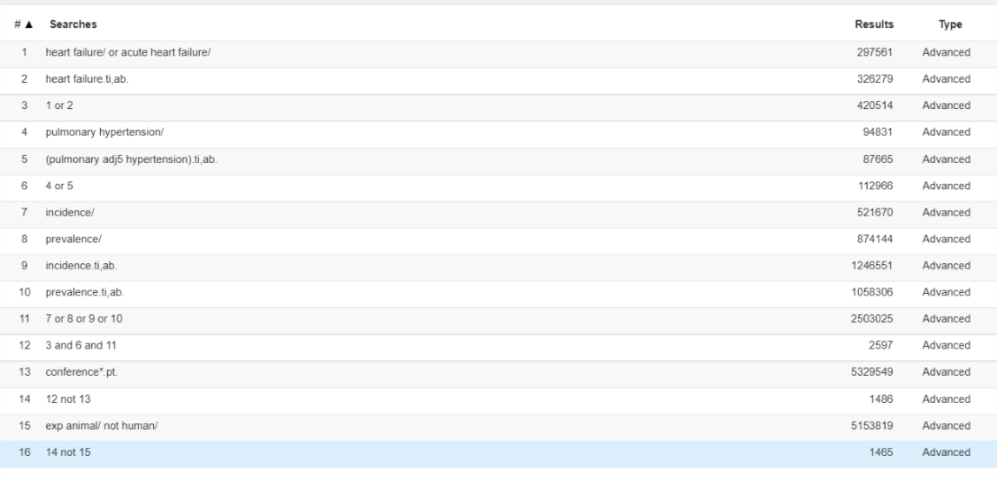
 

Medline


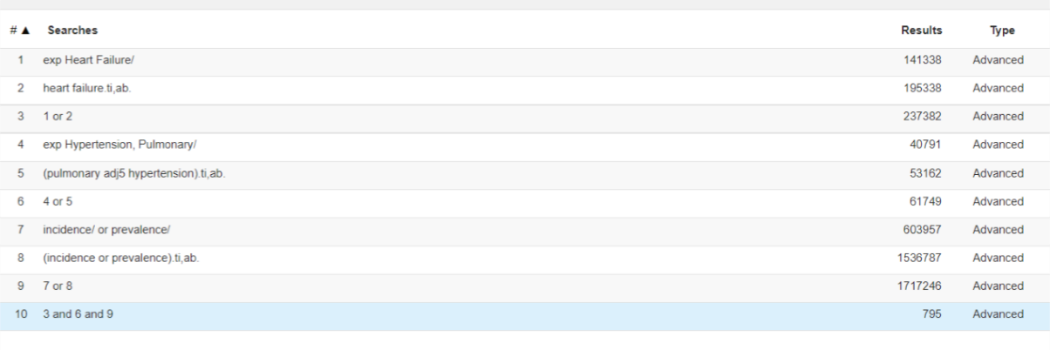
 

**Supplementary table 2. Quality assessment of the included papers using Johanna Briggs Institute Critical Appraisal Tool for systematic reviews**

| **Paper** | **1** | **2** | **3** | **4** | **5** | **6** | **7** | **8** | **9** |
| --- | --- | --- | --- | --- | --- | --- | --- | --- | --- |
| Ahmadi 2021 | No | Unclear | No | Yes | No | Unclear | Yes | Yes | Unclear |
| Ahmed 2020 | Yes | Unclear | No | Yes | Unclear | No | Yes | Yes | Unclear |
| Allison 2016 | Yes | Yes | Yes | Yes | Yes | No | Yes | Yes | Yes |
| Alqahtani 2019 | No | Yes | Yes | Yes | Unclear | No | No | Yes | Unclear |
| Amadi 2016 | No | Unclear | Yes | Yes | Unclear | Yes | Unclear | Yes | Unclear |
| Arnaert 2021 | Yes | Yes | Yes | Yes | Unclear | Unclear | No | Yes | Unclear |
| Auffret 2020 | Yes | Yes | Yes | Yes | Unclear | No | Unclear | Yes | Unclear |
| Benito-Gonzalez 2020 | Yes | Yes | No | Yes | Yes | Yes | Unclear | Yes | Yes |
| Bosch 2017 | Yes | Yes | Yes | Yes | Yes | Yes | Yes | Yes | Yes |
| Butler 1999 | Yes | Yes | Yes | No | Yes | Yes | Yes | Yes | Yes |
| Chakraborty 2022 | Yes | Yes | Yes | Yes | Yes | Unclear | Unclear | Yes | Yes |
| Choudhary 2014 | Yes | Yes | Yes | Yes | Yes | Unclear | Yes | Yes | Yes |
| Covella 2017 | Yes | Yes | Yes | Yes | Yes | Yes | Yes | Yes | Yes |
| Diaconu 2015 | No | Unclear | No | No | Yes | No | No | No | Yes |
| Faggiano 2000 | Yes | Yes | Yes | Yes | Yes | Yes | Yes | Yes | Unclear |
| Gerges 2015 | Yes | Yes | Yes | Yes | Yes | Yes | No | Yes | Yes |
| Guazzi 2017 | Yes | Yes | Yes | Yes | Yes | Yes | No | Yes | Yes |
| Haddad 2022 | Yes | Yes | No | Yes | Yes | Yes | No | Yes | Yes |
| Hsieh 2016 | Yes | Yes | Yes | Yes | Yes | Yes | No | Yes | Yes |
| Huang 2022 | Yes | Yes | Yes | Yes | Yes | Yes | Yes | Yes | Yes |
| Ibe 2016 | No | Yes | Yes | Yes | Unclear | Yes | Yes | Yes | Unclear |
| Jentzer 2022 | Yes | Yes | Yes | Yes | Yes | Yes | No | Yes | Yes |
| Kanumuri 2019 | Yes | Yes | No | Yes | Yes | Yes | Yes | Yes | Unclear |
| Karaye 2013 | Yes | Yes | No | Yes | Yes | Yes | Yes | Yes | Unclear |
| Khush 2009 | Yes | Unclear | Yes | Yes | Yes | Yes | Unclear | Yes | Yes |
| Kushimo 2019 | Yes | Yes | Yes | Yes | Unclear | Yes | Yes | Yes | Unclear |
| Lam 2009 | Yes | Yes | Yes | Yes | Yes | Yes | Yes | Yes | Yes |
| Lee 2022 | Yes | Yes | Yes | Yes | Yes | No | No | Yes | Yes |
| Leung 2010 | No | Yes | Yes | Yes | Yes | Unclear | No | Yes | Yes |
| Lima 2019 | Yes | Yes | Yes | Yes | Yes | Unclear | No | Yes | Unclear |
| Lin 2022 | Yes | Yes | Yes | Unclear | Yes | Unclear | Unclear | Yes | Yes |
| Liu 2020 | Yes | Yes | Yes | Yes | Unclear | Yes | Yes | Yes | Unclear |
| Lutsey 2022 | Yes | Yes | Yes | Yes | Yes | No | No | Yes | Yes |
| Mogollon 2008 | Yes | Unclear | No | No | Yes | Unclear | Unclear | Yes | Yes |
| Mutlak 2018 | Yes | Yes | Yes | Yes | Unclear | Yes | Yes | Yes | Unclear |
| Nakagawa 2020 | Yes | Yes | No | Yes | Unclear | Yes | Yes | Yes | Unclear |
| Nakamura 2019 | Yes | Yes | Yes | Yes | Yes | Yes | Unclear | Yes | Yes |
| Nkoke 2022 | Yes | Yes | No | Yes | Yes | Unclear | Yes | Yes | Yes |
| Pandey 2020 | Yes | Yes | Yes | Yes | Yes | Unclear | No | Yes | Yes |
| Pintalhao 2017 | Yes | Yes | Yes | Yes | Yes | Unclear | Unclear | Yes | Unclear |
| Raina 2015 | Yes | Unclear | Yes | No | Yes | Yes | Yes | Yes | Unclear |
| Raina 2015 | Yes | Yes | No | Yes | Unclear | Unclear | Yes | Yes | Unclear |
| Rifaie 2010 | Yes | Yes | Yes | Yes | Yes | Yes | Unclear | Yes | Yes |
| Santas 2019 | Yes | Yes | Yes | Yes | Yes | Yes | Yes | Yes | Unclear |
| Selvaraj 2017 | Yes | Unclear | Yes | Yes | Yes | Yes | Yes | Yes | Unclear |
| Shah 2014 | Yes | Unclear | Yes | Yes | Yes | Yes | Yes | Yes | Yes |
| Sobieszczanska-Malek 2014 | Yes | Yes | Yes | Unclear | Yes | Unclear | No | Yes | Yes |
| Stein 2012 | Yes | Yes | Yes | Yes | Yes | Yes | Yes | Yes | Yes |
| Straburzynska-Migaj 2007 | Yes | Unclear | No | Unclear | Yes | Unclear | Yes | Yes | Unclear |
| Torres-Macho 2012 | Yes | Unclear | Yes | Yes | Unclear | Yes | Yes | Yes | Unclear |
| Vanhercke 2015 | Yes | Yes | Yes | Yes | Yes | Yes | Unclear | Yes | Yes |
| VanWezenbeek 2022 | Yes | Yes | No | Yes | Unclear | Unclear | Yes | Yes | Yes |
| Wang 2014 | Yes | Yes | Yes | Yes | Yes | Yes | Yes | Yes | Yes |
| Zotter-Tufaro 2015 | Unclear | Unclear | Yes | No | Unclear | Unclear | Unclear | Yes | Unclear |

| 1 = Was the sample frame appropriate to address the target population? |
| --- |
| 2 =  Were study participants sampled in an appropriate way? |
| 3 = Was the sample size adequate? |
| 4 = Were the study subjects and the setting described in detail? |
| 5 = Was the data analysis conducted with sufficient coverage of the identified sample? |
| 6 = Were valid methods used for the identification of the condition? |
| 7 =  Was the condition measured in a standard, reliable way for all participants? |
| 8 = Was there appropriate statistical analysis? |
| 9 = Was the response rate adequate, and if not, was the low response rate managed appropriately? |

**Supplementary table 3. Complete data extracted from the included studies**

See separate file.

**Supplementary figure 1. Pooled prevalence of PH amongst individuals with HF pooled including Lima 2019, using the common and random effects model.** For studies reporting subgroup and overall estimates, only the overall estimate was included in this analysis which is indicated by (Overall). If no overall estimate was reported by the paper, the subgroup data was included in this analysis and the specific subgroup is indicated in brackets. Raina 2015a and Raina 2015b represents 2 different papers with separate patient cohorts published in the same year.


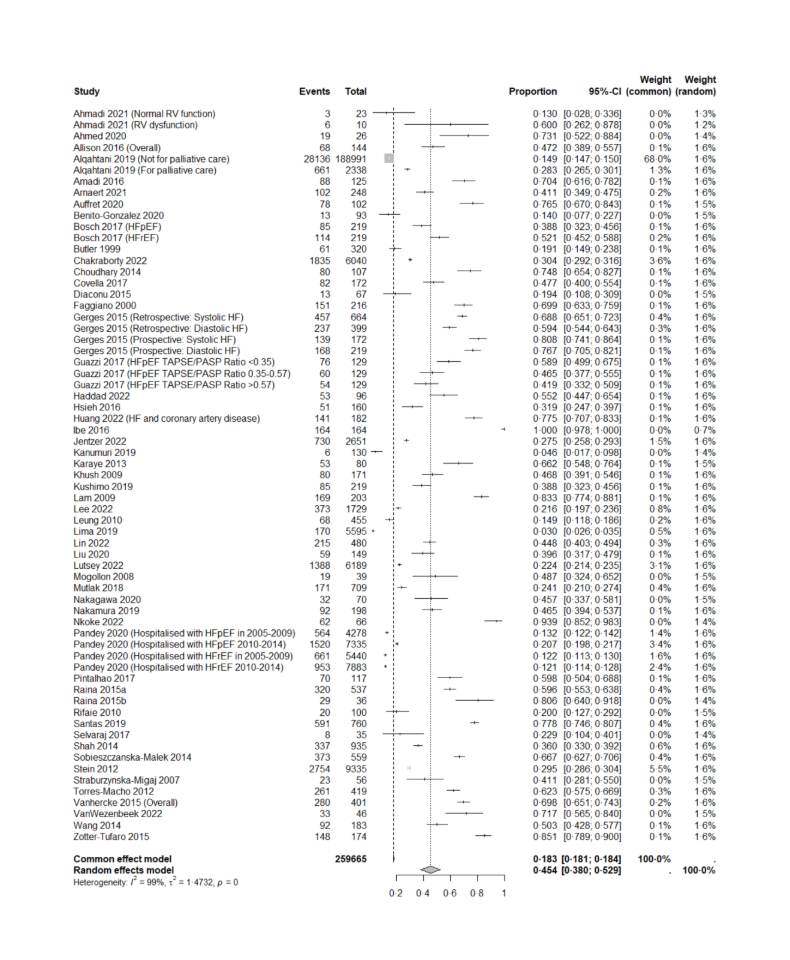


**Supplementary figure 2. Prevalence of PH amongst individuals with HF by diagnostic method excluding Lima 2019, using the common and random effects model.**


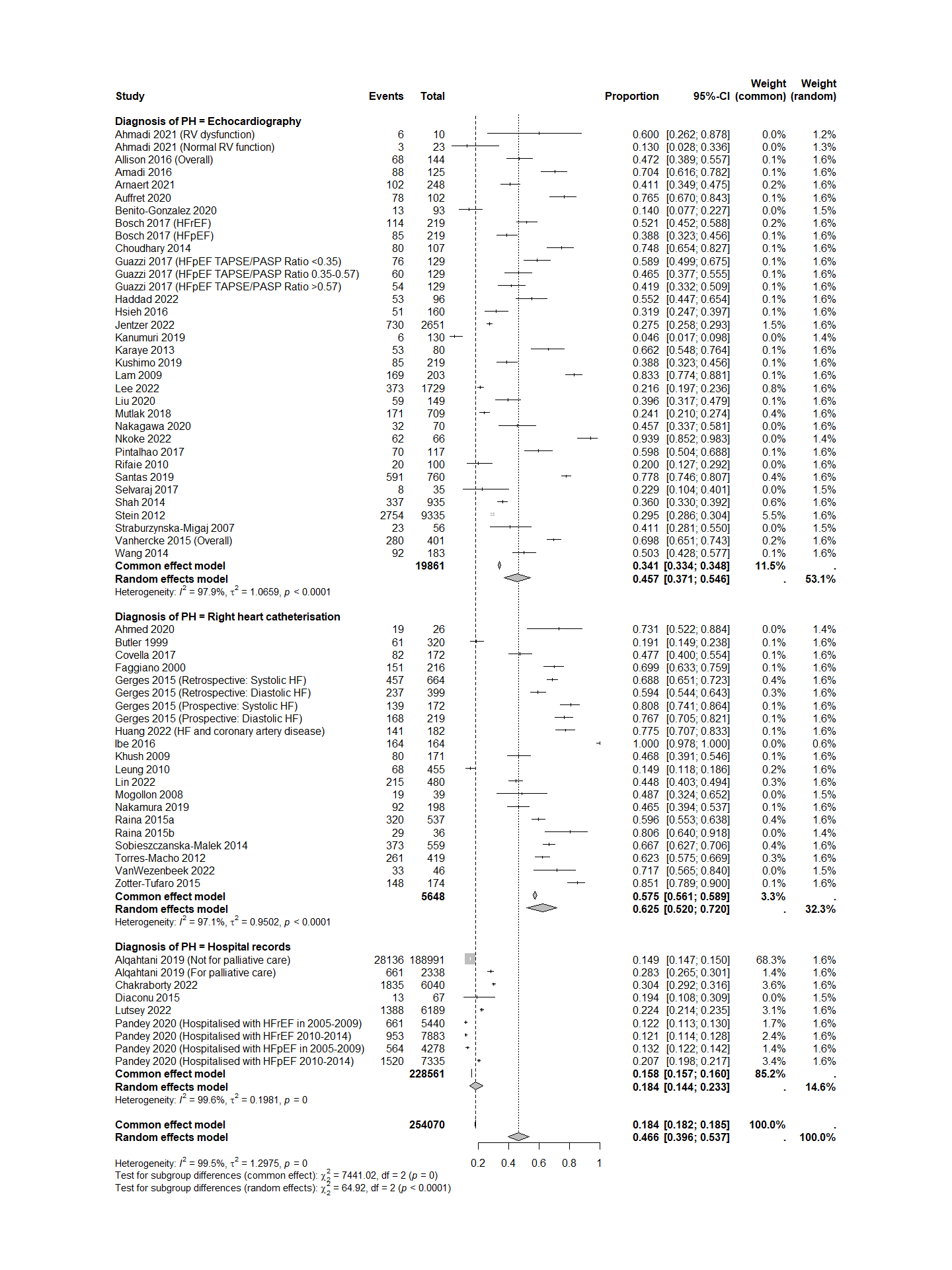


**Supplementary figure 3. Prevalence of PH in individuals with HF by study methodology.**


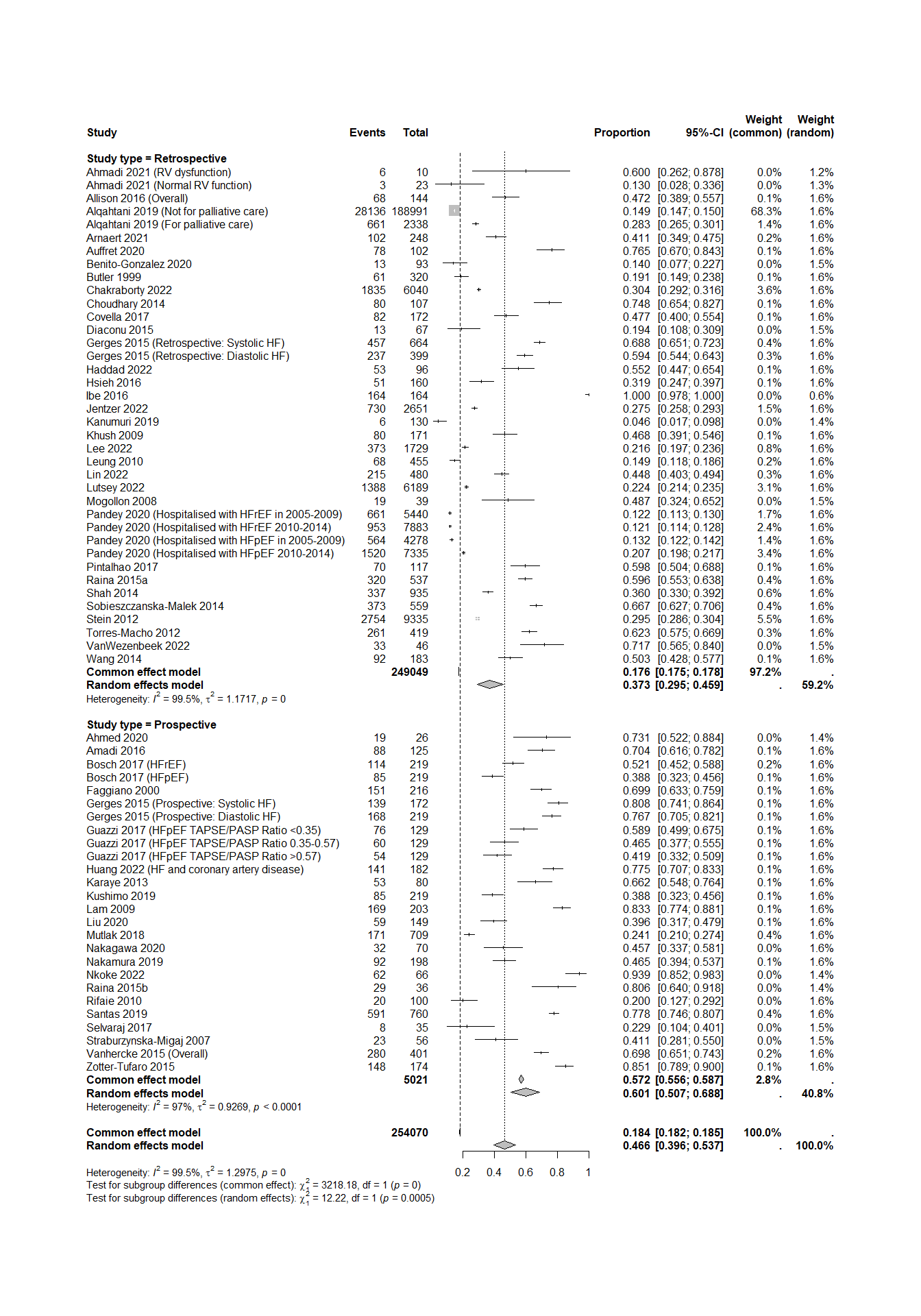


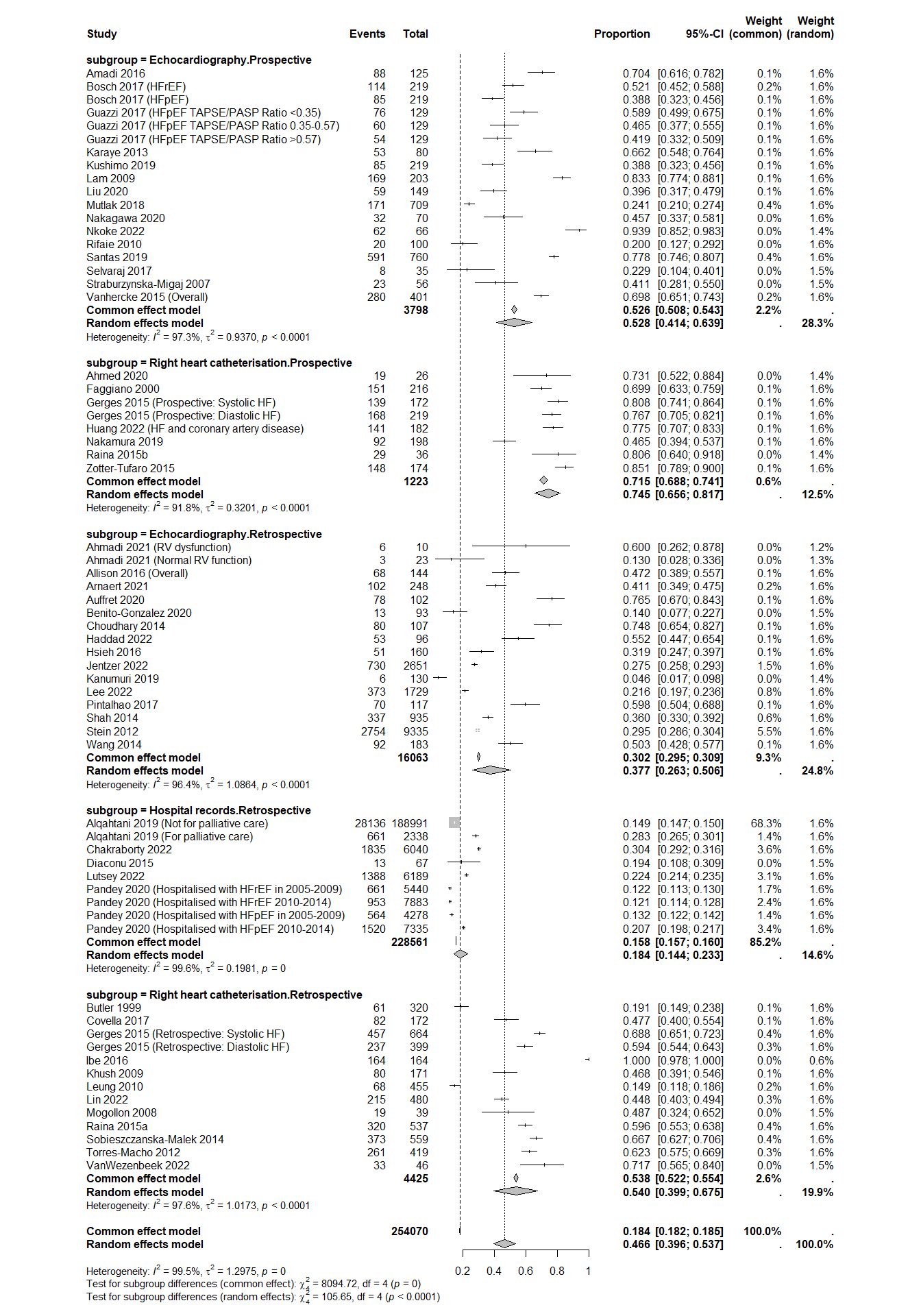
**Supplementary figure 4. Prevalence of PH in individuals with HF by study design (retrospective vs prospective) and diagnosis method.**

**Supplementary figure 5. Prevalence of PH in individuals with HF by ejection fraction.**


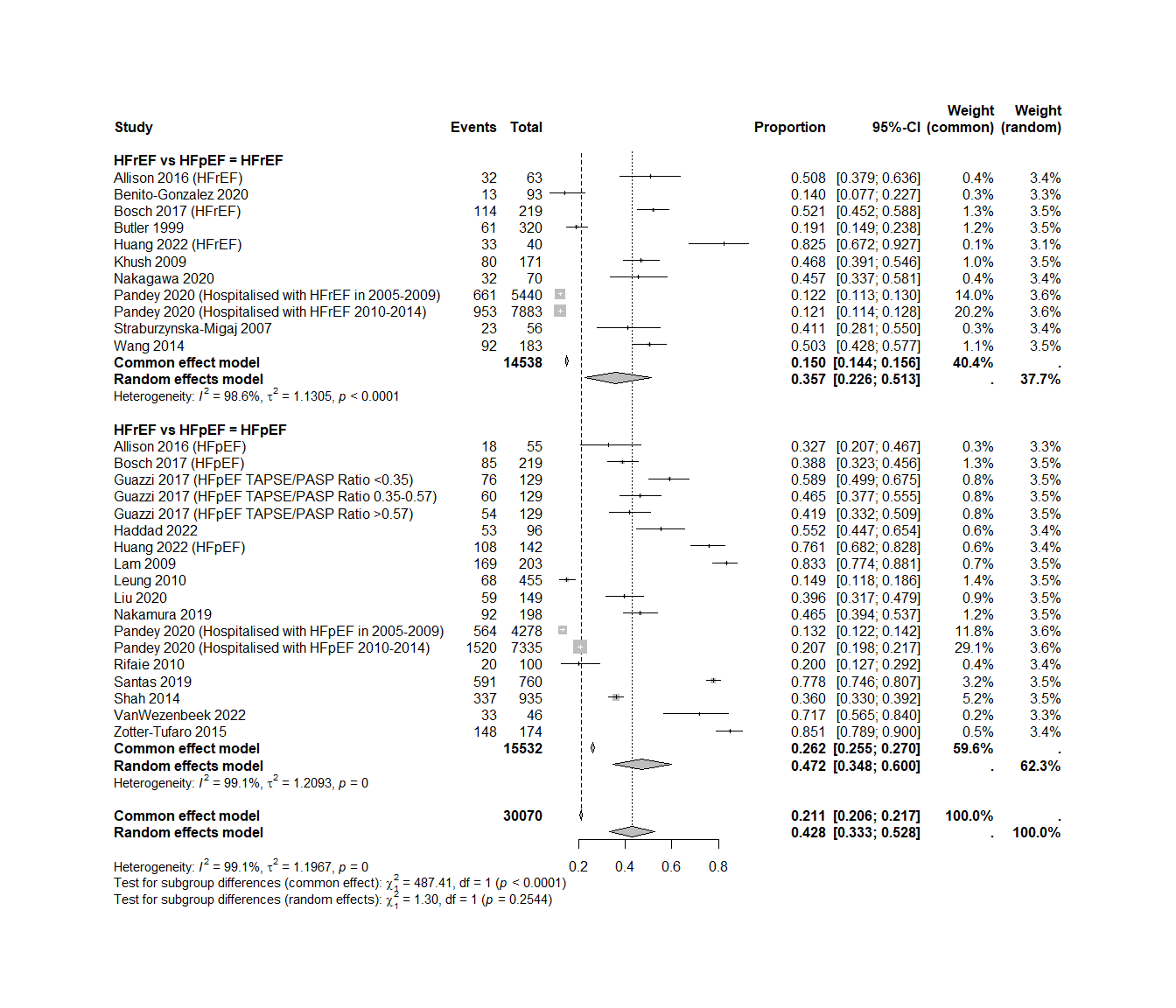


**Supplementary figure 6. Prevalence of PH in individuals with HF by ejection fraction and diagnostic method.**

RHC


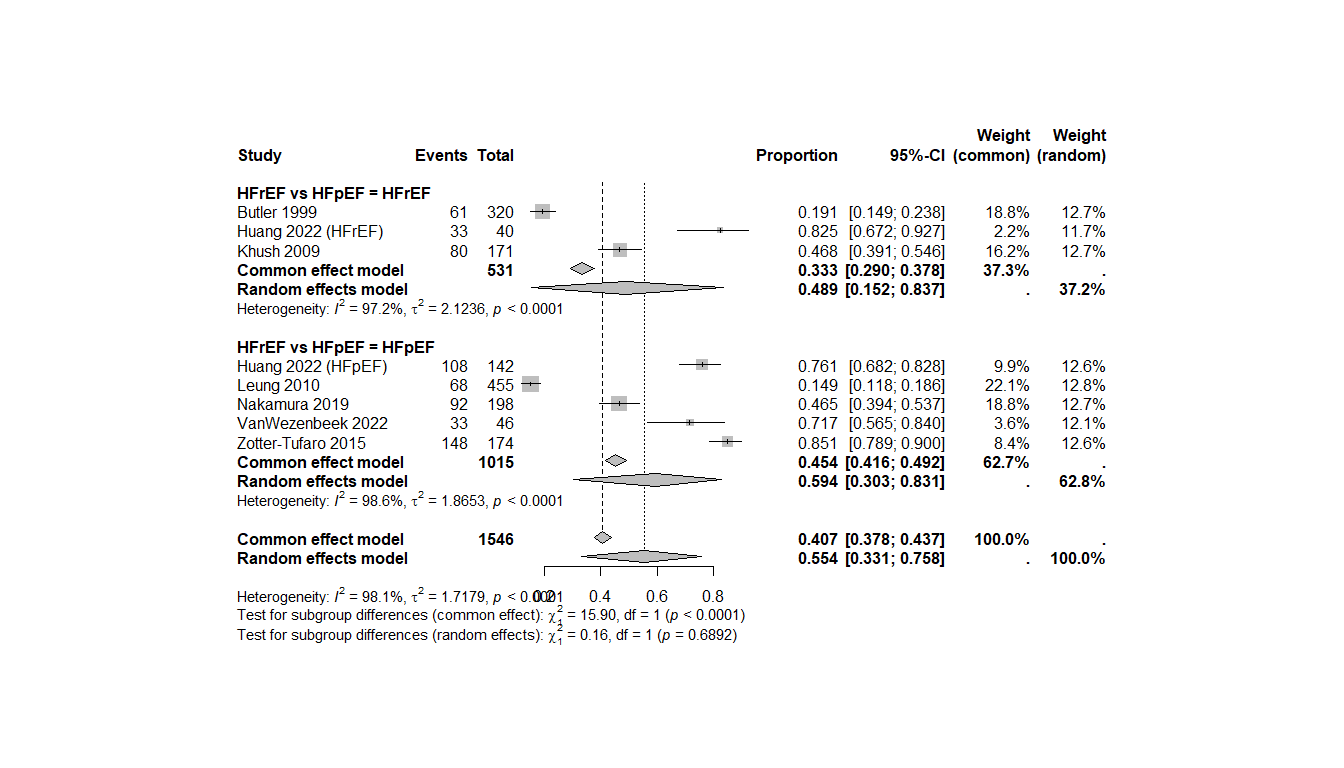


Echo


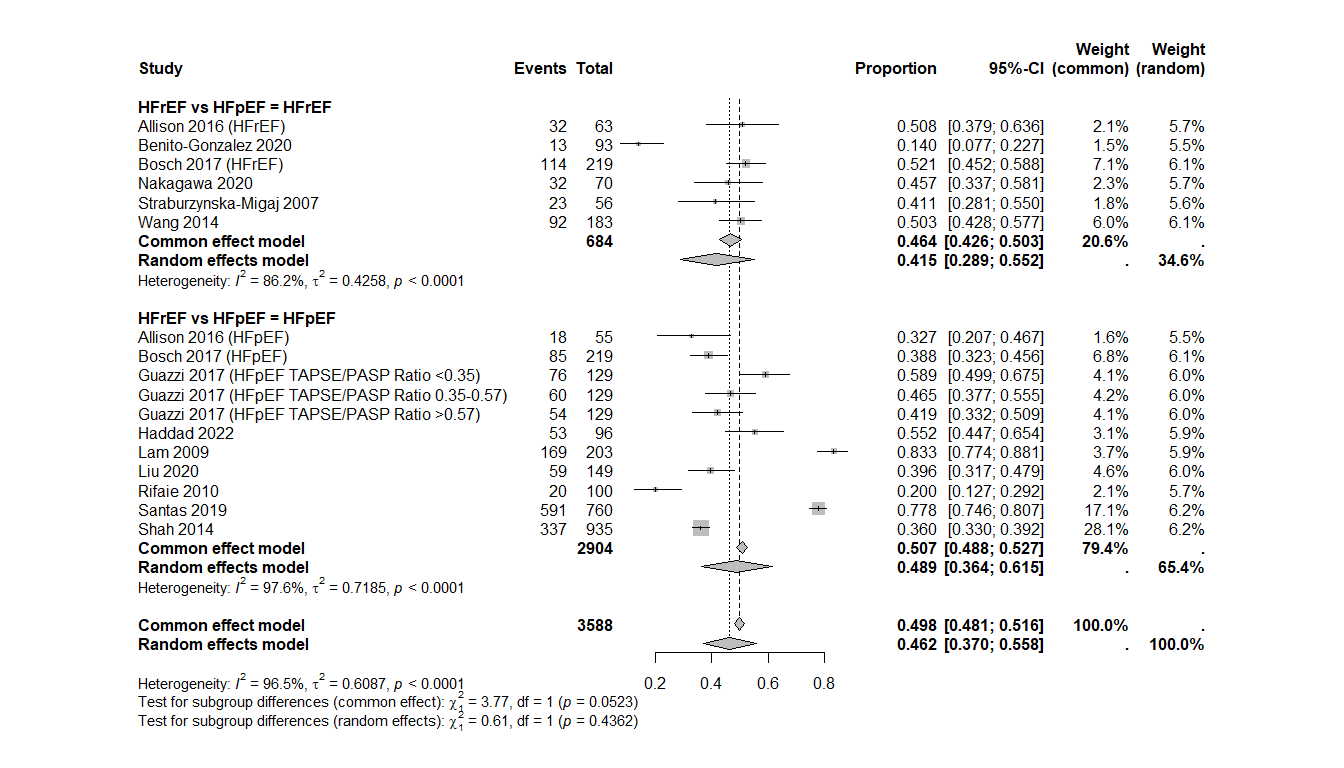


Hospital recorded data


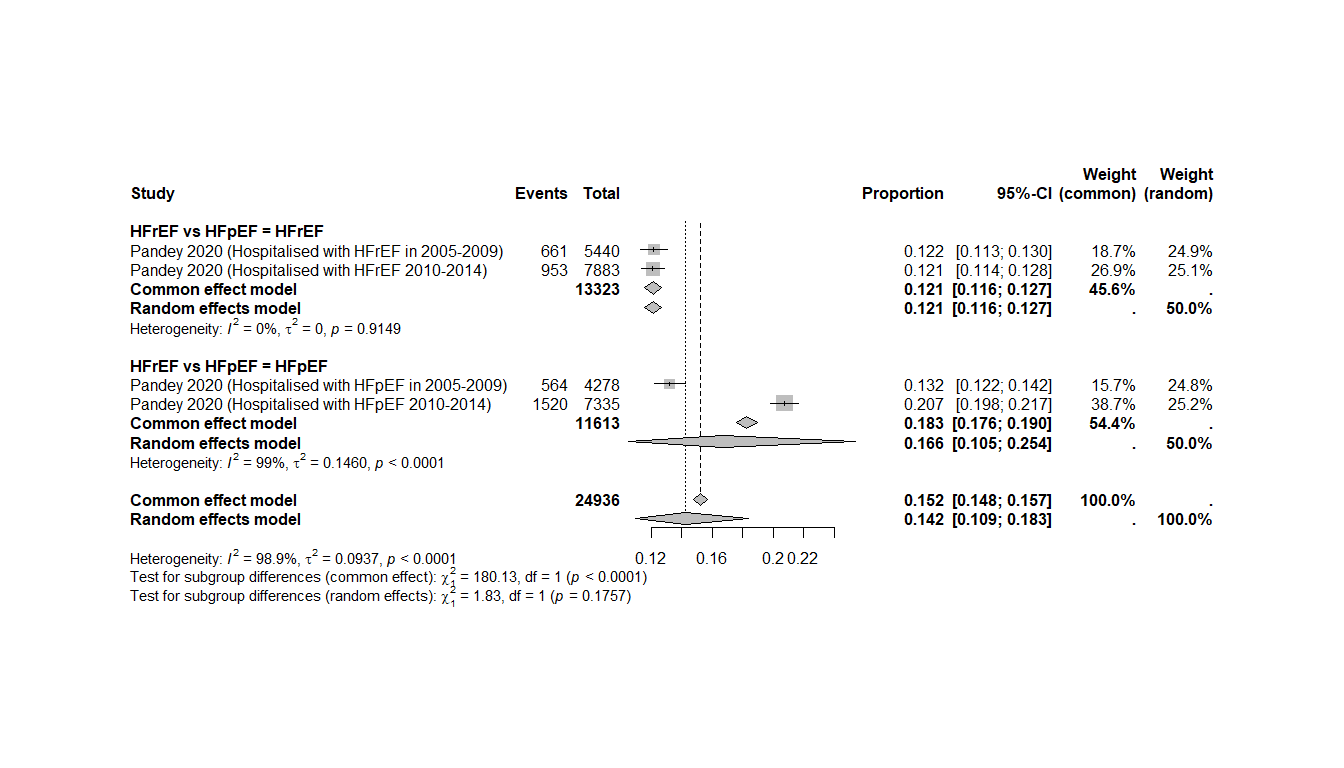


**Supplementary figure 7. Prevalence of PH in individuals with HF by location.**


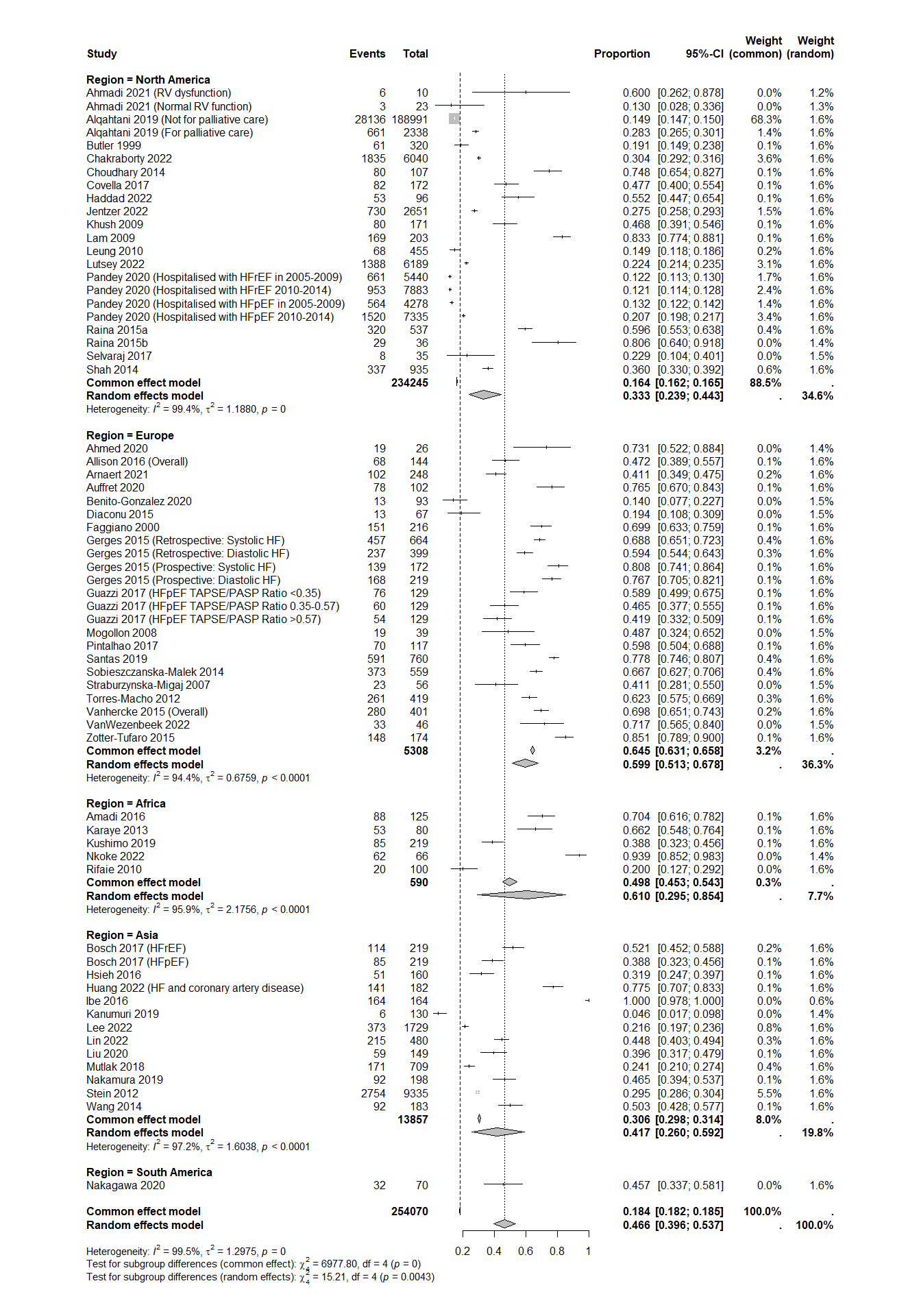


**Supplementary figure 8. Prevalence of PH in individuals with HF by country income classification.**


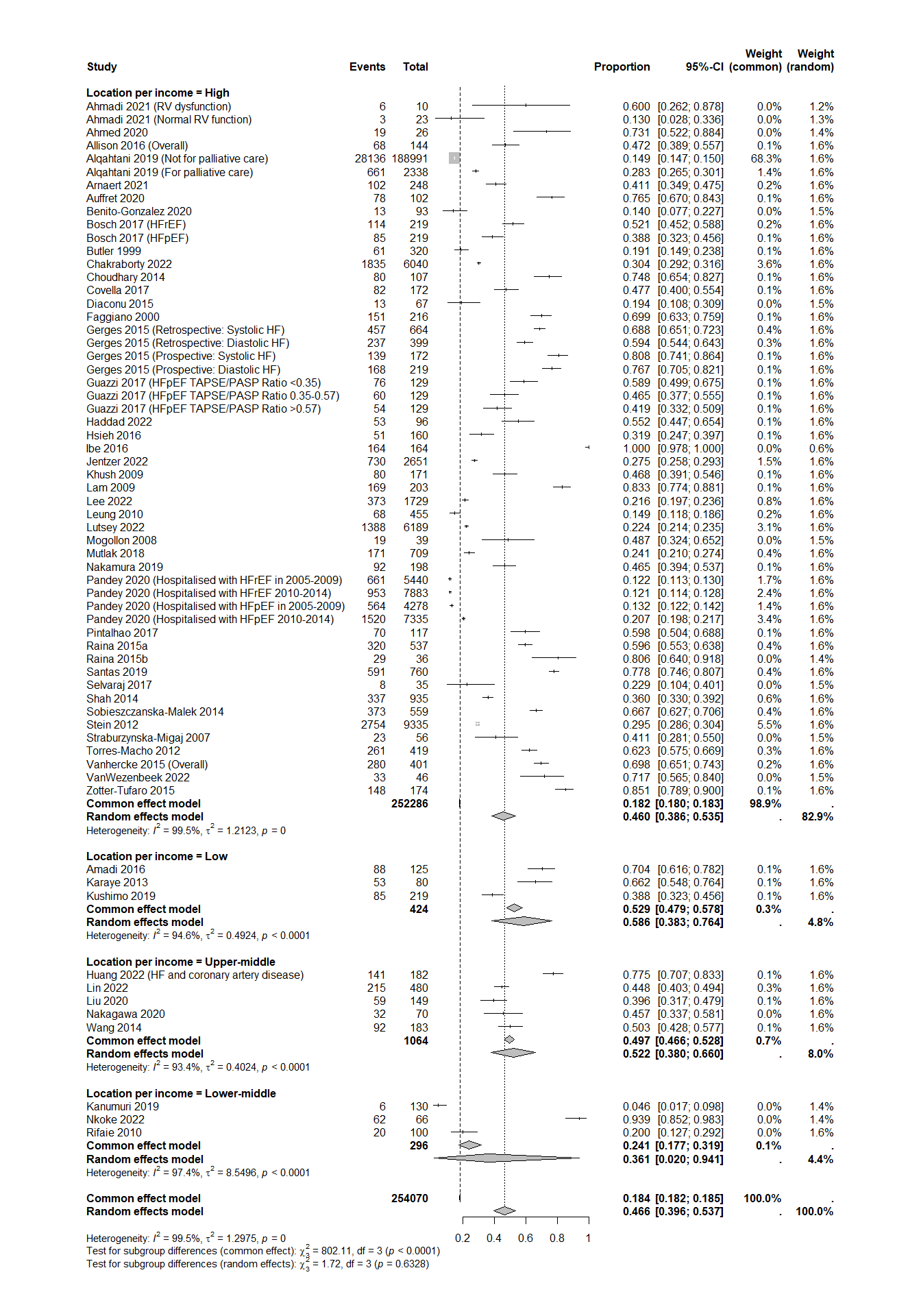


**Supplementary figure 9. Meta-regression of prevalence of PH in individuals with HF by proportion of participants with (A) mean age, (B) proportion with kidney disease, (C) proportion with atrial fibrillation, and (D) proportion with ischaemic heart disease**


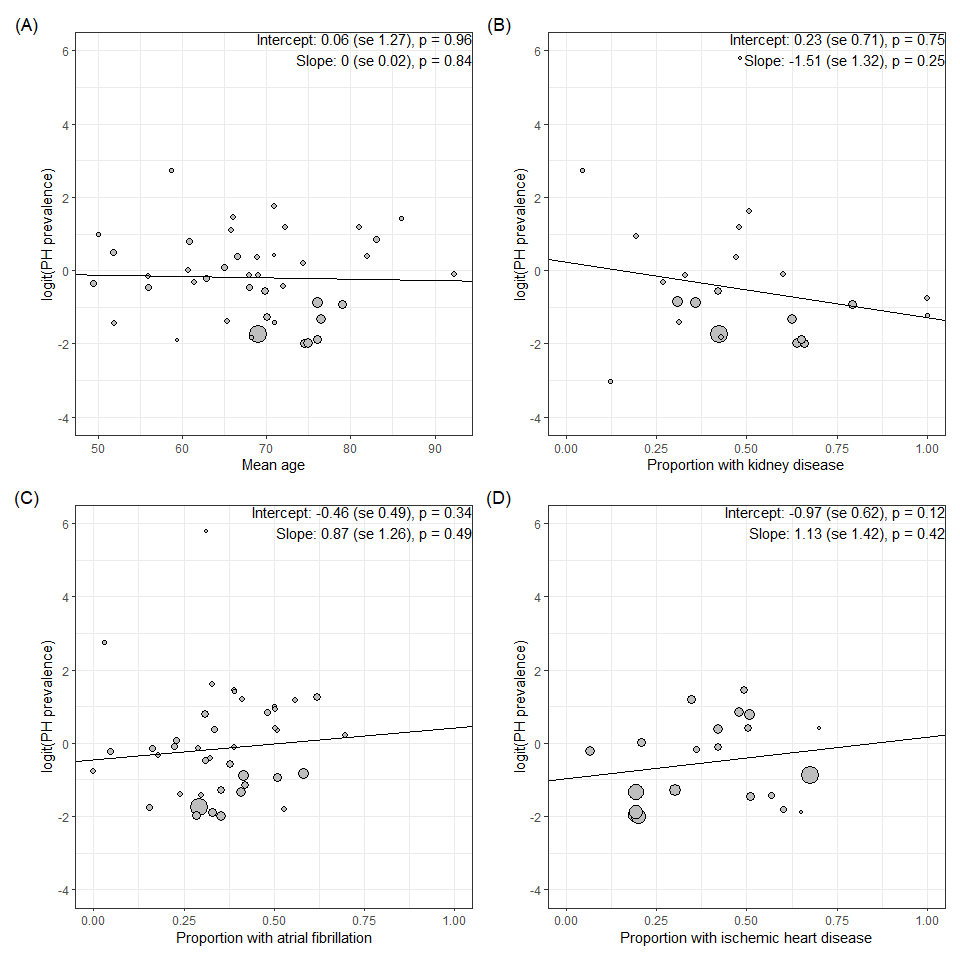

Supplement: Supplementary file 1 — Supplementary information. [file CLC-48-e70197-s002.docx]
